# An organic hole-transporting material spiro-OMeTAD doped with a Mn complex for efficient perovskite solar cells with high conversion efficiency

**DOI:** 10.1039/d1ra05906h

**Published:** 2021-10-05

**Authors:** Mohammed Elawad, Kingsley Igenepo John, Ahmed Mahmoud Idris, Li Yang, Yuan Gao

**Affiliations:** Faculty of Materials and Chemical Engineering, Yibin University 64400 Yibin China mohammed.toum@yahoo.com 2020100003@yibinu.edu.cn; Lab of Department of Pure and Applied Chemistry, College of Natural Sciences, Veritas University Abuja PMB 5171 Abuja Nigeria; Department of Chemistry, College of Chemistry and Life Science, Zhejiang Normal University (ZJNU) 688# Yingbin Road Jinhua Zhejiang 321004 P. R. China; Department of Chemistry, Faculty of Science, Omdurman Islamic University P. O. Box 382 Omdurman Sudan; State Key Laboratory of Catalysis, Division of Solar Energy, Dalian National Laboratory for Clean Energy, Dalian Institute of Chemical Physics, Chinese Academy of Sciences Dalian 116023 China

## Abstract

2,{2}′,7,{7}′-Tetrakis(*N*,*N*-di-*p*-methoxyphenylamine)-9,{9}′-spiro-bi-fluorene(spiro-OMeTAD) has often been used as a hole-transporting material (HTM) in mesoscopic perovskite solar cells (PSCs). However, its potential applications are limited due to its poor conductivity of approximately 10^−6^ to 10^−5^ cm^2^ V s^−1^ in pristine form, and this influences the stability and intrinsic hole conductivity of the device. In this work, a Mn complex [(Mn(Me-tpen)(ClO_4_)_2_^−^)]^2+^ is introduced as a p-dopant to improve the properties of spiro-OMeTAD-based PSCs, including the optical, electrical, conductivity, and stability properties. Interestingly, the use of spiro-OMeTAD with an optimum concentration (1.0% w/w) of Mn complex in mesoscopic PSCs achieves a remarkable power conversion efficiency of 17.62% with a high conductivity of 99.05%. Spiro-OMeTAD with Mn complex as a p-dopant under UV-vis spectroscopy shows a different peak at 520 nm, confirming that oxidation occurs upon the addition of the Mn complex. The enhanced efficiency of the PSCs may be attributed to an increase in the optical and electrical properties of the HTM in the spiro-OMeTAD doped Mn complex.

## Abstract 2

Mesoscopic PSCs utilizing spiro-OMeTAD with different concentrations of a Mn complex were obtained, and the PSC with the optimum Mn-complex concentration (1.0% w/w) achieved a remarkable power conversion efficiency of 17.62%.

## Graphical abstract

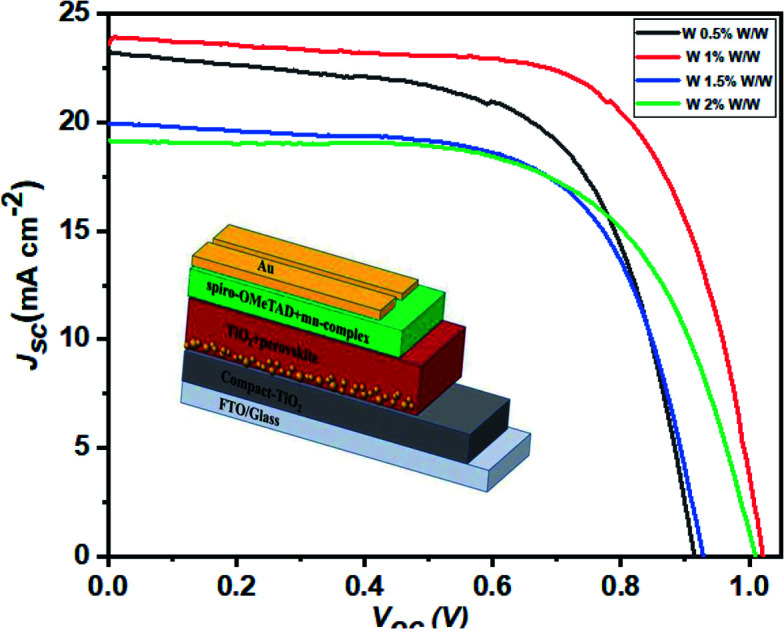

## Introduction

Nowadays solar cells are considered to be an alternative sustainable source of energy because they are clean, safe, and have constantly improving characteristics. However, studies are ongoing to create new strategies for improving the performance of solar cells to meet the increasing demand for a green and efficient alternative energy source.^1^ For example, perovskite solar cells (PSCs) have been subjected to component engineering, a deposition technique to manufacture high-quality perovskite films, device layer designing, increasing of layer conductivity, and stability enhancements. Thus far, different techniques have been developed, among which, use of photovoltaics, which directly converts solar energy into electricity, has attracted more attention.^2–5^ The PSCs have remarkable properties: good diffusion length, high charge carrier mobility, high absorption coefficient, and broad spectral absorption range, and they show great promise for use as solar cell devices with great light-absorbing materials. In the past years, PSCs with over 25% power conversion efficiency (PCE) have been developed.^6^

The hole-transporting material (HTM) in PSCs minimizes recombination by extracting the photogenerated holes, and then transporting them to the metal electrode.^7^ Organic and inorganic semiconductors have also been employed as HTMs in PSCs.^8–11^ For example, Chen *et al.* developed organic HTMs with two hole-extraction materials (HEMs) to fabricate efficient planar inverted (p–i–n) perovskite solar cells (PVSCs). The first and second HEMs were TPP-OMeTAD, and TPP-SMeTAD, which achieved PCEs of 14.6% and 16.6%, respectively.^12^ Zhang *et al.* inserted a thin insulating polymer (PMMA or PS) as a passivation layer between the perovskite and the p-doped HTMs in planar inverted PVSCs to improve the *V*_oc_ and fill factor (FF). The PCEs were increased by use of passivation trap-assisted interfacial recombination and this inhibited the direct shunting from the p-type doping of the HTMs. A highly efficient PVSC with a steady-state achieved a PCE of 20.3%.^13^ Several types of HTM have been designed and are they are named based on their key components. Examples include conjugated donor–acceptor small molecule (TPA)-based, carbazole-based, tetrathiafulvalene, and pentacene derivatives.^14–20^ Among these, spiro-OMeTAD has been demonstrated to exhibit the highest PCE of over 20%.^21^ In PSCs, small molecules such as 2,{2}′,7,{7}′-tetrakis(*N*,*N*-di-*p*-methoxyphenylamine)-9,{9}′-spiro-bi-fluorene (spiro-OMeTAD) were introduced as HTMs, but were limited as a result of their low conductivity (approximately 10^−6^ to 10^−5^ cm^2^ V s^−1^) in pristine form.^7,22,23^

Recently, most studies have focused on using a small organic molecule spirobifluorene-based bulky molecule (spiro-OMeTAD) in HTM, which has so far displayed the best performance since its discovery in 1998.^24,25^ At first, it was established for use in solid-state dye-sensitized solar cells (DSSCs), however, a PCE of only 0.74% was achieved without any dopant, and it increased to 2.56% by adding 4-*tert*-butylpyridine (TBP) and lithium bis(trifluoromethanesulfonyl)imide (Li-TFSI) additives.^26^ Gratzel and co-workers increased the conductivity and efficiency of spiro-OMeTAD using a Co(iii) compound (FK102) as an additive and achieved a PCE of 7.2% at one sun illumination.^24^ Furthermore, different Co(iii) complexes are still utilized as additives and are being investigated to improve the hole mobility and *V*_oc_ of spiro-OMeTAD and other complexes such as p-type dopants. Spiro-OMeTAD has been used in PSCs as a HTM, and a PCE of 18.5% was obtained.^10,12,16–36^ Many p-type dopants in HTM materials have been reported,^29,32^ and Co(iii) complexes (FK102) have been used with concentrations of 1.0%, 1.6%, and 2.2% and have achieved PCEs of 4.3%, 5.3%, and 5.6%, respectively.^10^ Tris(2-(1*H*-pyrazol-1-yl)-4-*tert*-butylpyridine)cobalt(iii)tris-(bis(trifluoromethylsulfonyl)imide) FK209, bis(2,6-di(1*H*-pyrazol-1-yl)pyridine)cobalt(iii)tris-(bis(trifluoromethylsulfonyl)-imide) FK269, and tris[2-(1*H*-pyrazol-1-yl)pyrimidine]cobalt(iii)tris-[bis(trifluoromethylsulfonyl)-imide] MY11 exhibited PCEs of 6.0%, 6.0%, and 12%, respectively.^36,37^ The 1,1,2,2-tetrachloroethane was used as a solvent and additive for spiro-OMeTAD in solid-state DSSCs which exhibited a PCE of 7.7%.^38^ A spiro-OMeTAD-based HTM with silver salt (Ag-TFSI) as a p-dopant achieved a PCE of 12.0%.^39^ As p-dopants with P3HT in HTM-based PSCs, single-walled carbon nanotubes, a conductive carbon material (graphdiyne), and bamboo-structured carbon nanotubes exhibited PCEs of 15.3%, 14.58%, and 8.3%, respectively.^30,33^ The F4-TCNQ was used as a p-dopant in spiro-OMeTAD with a PCE of 5.44%,^40^ and PTAA-based PSCs achieved a PCE of 17.5%.^31,32^ Furthermore, spiro-OMeTAD TBP, and Li-TFSI were utilized with, FK209, and LD29 as HTM-based PSCs, which exhibited PCEs of 18.25% and 18.4%, respectively.^17^ Similarly, spiro-OMeTAD complexes of p-dopants of copper salts (cuprous iodide or cuprous thiocyanate) and copper(ii)bis[bis(trifluoromethylsulfonyl)imide] [Cu(bpcm)_2_] and copper(ii)bis[bis (trifluoromethyl-sulfonyl)imide] [Cu(bpm)_2_], achieved PECs of 18.02%, 12.8%, and 18.5%, respectively.^41–43^ Bi *et al.* fabricated mesoscopic PSCs with doped spiro-OMeTAD-based HTM in a configuration of FTO/compact TiO_2_/mesoporous TiO_2_/perovskite/spiro-OMeTAD/Au architecture, which achieved a spectacular *V*_oc_ of 1.16 V and exhibited a PCE of 20.8%.^21^ The spiro-OMeTAD highest occupied molecular orbital (HOMO) level varies a lot under different doping concentrations. The HOMO level is about −5.16 eV before doping and becomes about −5.22 eV after doping by the p-dopants, which is slightly deeper. Normally, the HOMO level of the inert spiro-OMeTAD (which is not being doped) in dichloromethane (CH_2_Cl_2_) is measured by cyclic voltammetry (CV). However, the value changes after being deposited into a thin film (the HOMO level of a solid-state thin film can be measured by ultraviolet photelectron spectroscopy (UPS), but the value showed some discrepancy when compared with the CV results measured in solution).^44^

**Fig. 1:**
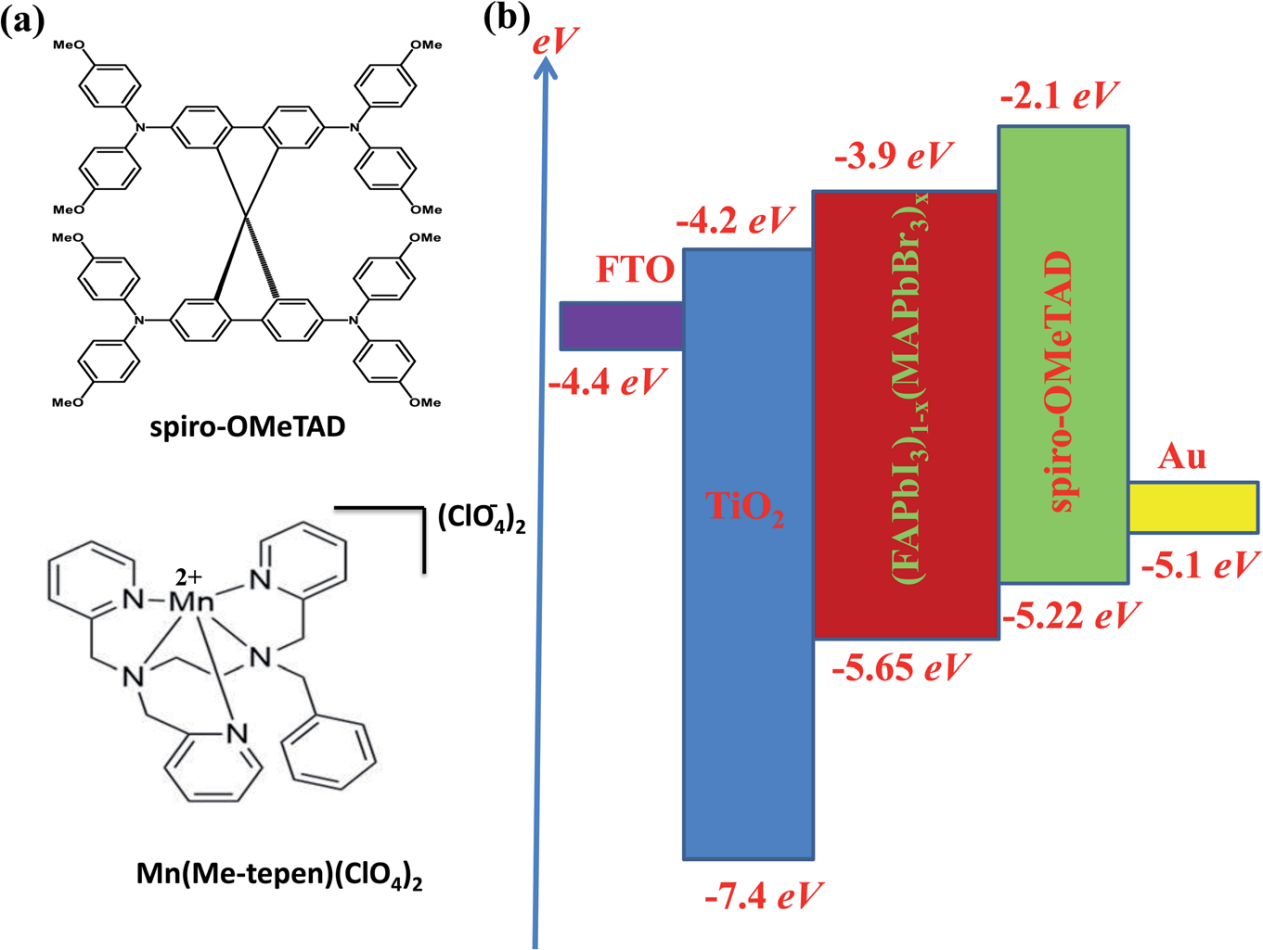
(a) The chemical structures of spiro-OMeTAD and the Mn complex. (b) The energy level diagram of the suggested solar cell.

Herein, for the first time [(Mn(Me-tpen)(ClO_4_)_2_^−^)]^2+^ (Mn complex) is successfully introduced as an effective additive for use with spiro-OMeTAD, as shown in Fig. 1a. The redox potential of the Mn complex and the HOMO energy levels of the various spiro-OMeTADs have improved the one-electron oxidation reaction. The increasing of the spiro-OMeTAD^+^ oxidized species into spiro-OMeTAD doped Mn complex solutions improved the conductivity in the films.

## Results and discussion

### Cyclic voltammetry

Previous studies have shown the cyclic voltammetry results for the redox potential of spiro-OMeTAD at 0.02 V *versus* Fc^+^/Fc.^45^

**Fig. 2:**
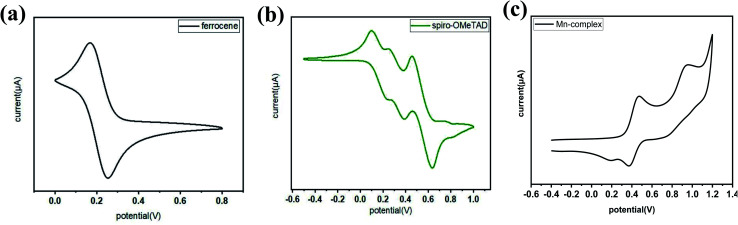
Cyclic voltammetry studies in the range of 0.0–1.1 V of (a) ferrocene, (b) spiro-OMeTAD, and (c) the Mn complex Mn(ii)/Mn(i) *versus* Fc^+^/Fc.

However, in the present study, the redox potential of the Mn complex was 0.32 V *versus* Fc^+^/Fc (Fig. 2a). The driving force of the Mn complex was more than the 300 mV of the dopant, which was sufficient for the complete one-electron extraction, as shown in Fig. 2b and c. The electron movement from the HOMO of spiro-OMeTAD to the Mn complex enhanced the production of hole carriers in spiro-OMeTAD, which improved the conductivity of spiro-OMeTAD.^46–49^

### UV-vis spectroscopy

In this study, the Li-TFSI/[(Mn(Me-tpen)(ClO_4_)_2_^−^)]^2+^/TBP was successfully used to p-dope spiro-OMeTAD, and the resultant complex attained a stabilized PCE of greater than 17%. The TA neutral absorption peak of spiro-OMeTAD appeared at 350 nm. Upon adding the Mn complex as a dopant to the spiro-OMeTAD solution, the films formed showed a decrease in the absorption peak at 350 nm and the appearance of two new absorption features at 400 nm and 520 nm. The latter peaks were observed to increase with increasing dopant concentrations, and the single peak decreased, as shown in Fig. 2a.^24^ The [(Mn(Me-tpen)(ClO_4_)_2_^−^)]^2+^ (Mn complex) p-doping of spiro-OMeTAD is demonstrated in Fig. 1a. The increased Mn complex in the solution caused more oxidation of the spiro-OMeTAD in conjunction with the Mn complex reduction, which is indicated by the curve of Mn complex-doped spiro-OMeTAD at 520 nm.^50^ Conversely, the UV-vis investigation of spiro-OMeTAD doped Mn(ClO_4_)_2_ solution did not display any curve at 520 nm related to the oxidized spiro-OMeTAD as shown in Fig. 4.

### Conductivity

**Fig. 3:**
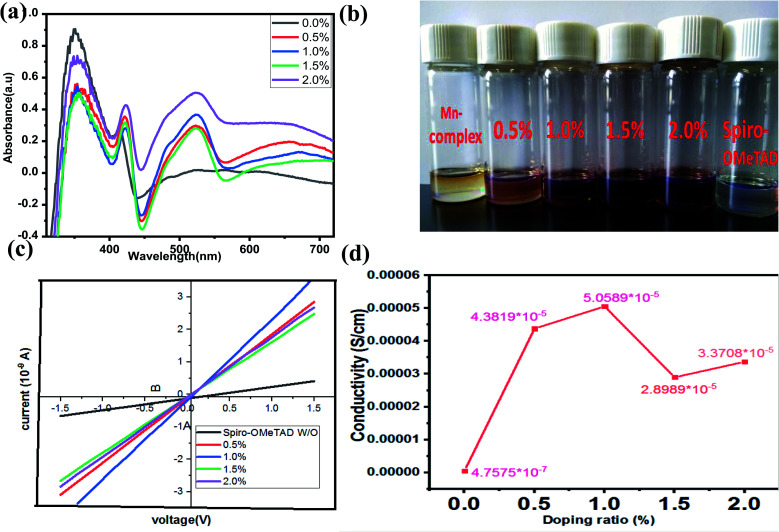
(a) The UV-vis absorption spectra of the Mn complex solution and spiro-OMeTAD solutions with different concentrations of Mn complex. (b) Solutions of spiro-OMeTAD with the Mn complex at different concentrations (0%, 0.5%, 1.0%, 1.5%, and 2.0%). (c) Current *versus* voltage curves and (d) a plot of conductivity *vs.* doping concentration for glass/compact TiO_2_/HTM/Au hole-only devices; linear fits to these curves are used to measure resistance and calculate the conductivity of different concentrations of Mn complex doped spiro-OMeTAD as the HTM.

The conductivity was measured using the equation *σ* = *L*/(*Rμd*), where *L* is the channel length, *R* is the film resistance calculated from gradients of the curves, *μ* is the channel width, and *d* is the film thickness, as shown in Fig. 3a. The undoped spiro-OMeTAD complex showed a conductivity of 4.7575 × 10^−7^ Ω cm^−2^, which was increased after the Mn complex p-doping, resulting in a concentration of more than 99.05% and conductivity of about 5.0589 × 10^−5^ Ω cm^−2^. The *J*–*V* curve conductivity measured for the p-doped Mn complex was 6.109 Ω cm^−2^, whereas that of the undoped spiro-OMeTAD complex was 2.37 Ω cm^−2^, resulting in a higher FF (1.0% w/w), which was higher than that for the other concentrations (0%, 0.5%, 1.5%, and 2.0%) used in this work. The effects of other concentrations of the undoped spiro-OMeTAD complex were determined and the results were compared with those of 1.0% (w/w) undoped spiro-OMeTAD complex and the conductivity was found to be 4.3819 × 10^−5^. The bulk conductivity increased by adding Mn complex to spiro-OMeTAD solutions of different concentrations, as mentioned previously and shown in Fig. 3b.

**Fig. 4:**
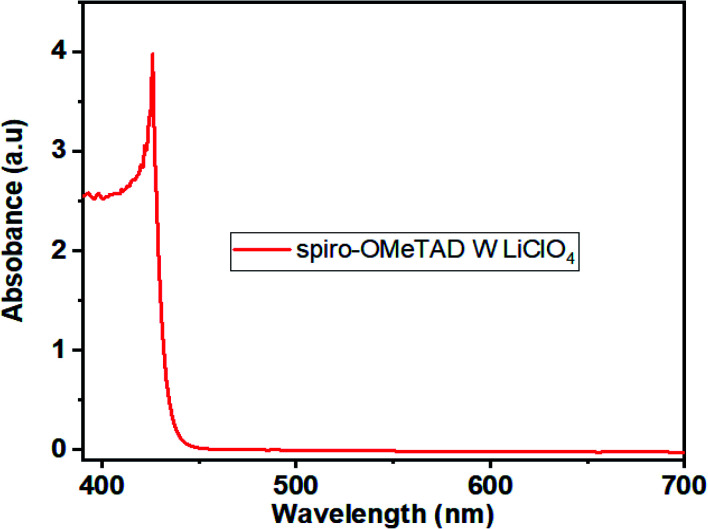
(a) The UV-vis absorption spectrum of spiro-OMeTAD doped Mn(ClO_4_)_2_ solution.

### Photovoltaic performances with the spiro-OMeTAD-doped Mn complex as the HTM

**Fig. 5:**
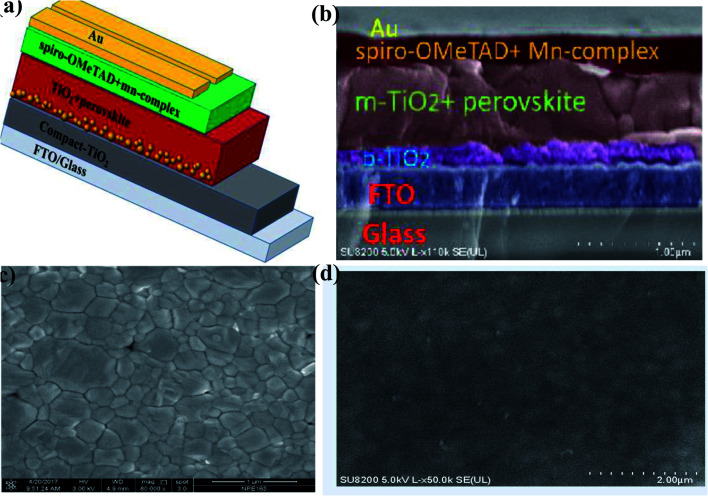
(a) The architecture of the (FAPbI_3_)_0.85_(MAPbBr_3_)_0.15_ containing solar cells. (b) A high-resolution SEM cross-sectional image of a PSC with the spiro-OMeTAD:Mn complex as the HTM. The layers are colored for better visibility. Top views of (c) perovskite film and (d) the spiro-OMeTAD:Mn complex film.

The PSC mesoscopic structured spiro-OMeTAD-doped Mn complex with different concentrations of Mn complex were fabricated. The device architecture of the cross-sectional SEM is shown in Fig. 5a and b. The PSC solar cells were designed with structure of glass/FTO/compact-TiO_2_ (∼30–40 nm)/mesoporous-TiO_2_ (∼200 nm)/(FAPbI_3_)_0.85_(MAPbBr_3_)_0.15_/HTM/Au. The mixed-cation perovskite light absorber (FAPbI_3_)_0.85_(MAPbBr_3_)_0.15_ was prepared as reported previously in the literature.^21,51^ Approximately 600 nm-thick perovskite crystals were constructed inside the scaffold layer, and more crystals created a capping layer at the top of the scaffold layer. The spiro-OMeTAD (∼200–216 nm)-doped Mn complex with different concentrations (0.5%, 1.0%, 1.5%, and 2.0%) was deposited on the top of the perovskite layer as the HTMs. The top-view SEM image has a uniform layer of spiro-OMeTAD:Mn complex that covered the perovskite layer well, as shown in Fig. 5c and d.

**Fig. 6:**
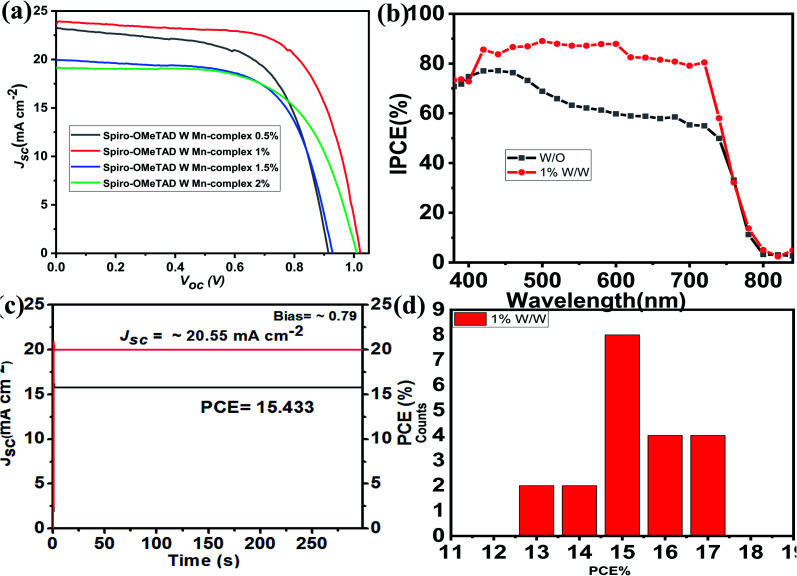
(a) The *J*–*V* curves of devices with different concentrations (0.5%, 1.0%, 1.5%, and 2.0%). (b) The IPCE values of spiro-OMeTAD without/with the Mn complex. (c) The current stability for spiro-OMeTAD doped with the Mn complex. (d) The PCEs for 20 PSCs with the Mn complex.

The current density–voltage (*J*–*V*) characteristics of the PSC devices based on spiro-OMeTAD-doped Mn complex with different concentrations of doping (0.5%, 1.0%, 1.5%, and 2.0%) as HTMs were measured under 100 mW cm^−2^ illumination (AM 1.5 G), and the results are displayed in Fig. 6a, and Table 1. The results for the devices prepared with different concentrations of spiro-OMeTAD-doped Mn complex, measured under AM 1.5 simulated sunlight (100 mW cm^−2^ irradiance) are shown in Table 1. The use of spiro-OMeTAD-doped Mn complex-based HTMs with different concentrations of Mn complex in PSCs has an effect on the overall efficiency, as shown by the improvement of the *J*_sc_ and FF results. The spiro-OMeTAD-doped Mn complex under ideal conditions (1.0% w/w) exhibited a maximum PCE of 17.62%, with a *V*_oc_ of 0.9367 V, a *J*_sc_ of 23.886 mA cm^−2^, and an FF of 0.78. The present study demonstrated the highest PCE for the spiro-OMeTAD-doped Mn complex-based PSCs used as an HTM. For the PSCs with an Mn complex concentration of 1.8% (w/w) doped spiro-OMeTAD, the higher FF of 0.78 was due to the additional charge carriers that were produced by the p-dopant, which improved the conductivity of the hole transport layer of the film. The spiro-OMeTAD-doped Mn complex (1.0% w/w) was used as the ideal concentration for subsequent experiments.

**Table 1:** Photovoltaic parameters of PSCs based on spiro-OMeTAD without/with the Mn complex as the HTM with different concentrations of Mn complex measured under 100 mW cm^−2^ illumination (AM 1.5 G)^a^

Doping ratio (% w/w)	*V*_oc_ (V)	*J*_sc_ (mA cm^−2^)	FF	PCE (%)
0.0	0.85	10.32	0.31	3.73
0.5	0.978	21.397	0.67	14.15
1.0	0.936	23.88	0.78	17.62
1.5	0.977	19.15	0.74	13.76
2.0	1.009	19.118	0.64	12.29

a *V*_oc_ = open circuit voltage, *J*_sc_ = open circuit current, PCE = power conversion efficiency.

The IPCE spectra of the undoped spiro-OMeTAD and spiro-OMeTAD-doped Mn complex-based PSCs as HTM are shown in Fig. 6b. These two PSC devices showed wide spectral responses at wavelengths greater than 800 nm. For comparison, the IPCE of undoped spiro-OMeTAD and spiro-OMeTAD-doped Mn complex-based PSCs over the whole region were measured, and the results obtained agree with the *J*–*V* measurement (IPCE spectra integrated *J*_sc_ was 23.43 mA cm^−2^, which matching the *J*–*V* results slightly). The PSCs with spiro-OMeTAD doped Mn complex under a bias voltage of 0.78 for over 300 s under one sun illumination (AM 1.5 G) achieved a steady-state efficiency of 15.433% and a current density of 20.55 mA cm^−2^ during testing, as shown in Fig. 5c. Differences in spiro-OMeTAD-doped Mn complex-based PSCs showed remarkably enhanced IPCE over the entire region in the range of 350–750 nm, yielding a substantial increase of over 80%.

The performance of the spiro-OMeTAD-doped Mn complexes with other doping concentrations were measured under identical conditions, and a peak PCE of 14.15% was obtained, with a *V*_oc_ of 0.978 V, a *J*_sc_ of 21.397 mA cm^−2^ and an FF of 0.67, as shown in Fig. 6a. The *J*_sc_ and FF of the PSCs based on other concentrations of the spiro-OMeTAD-doped Mn complex were compared with undoped spiro-OMeTAD, as shown in Table 1. The enhancement of the photovoltaic parameters of the spiro-OMeTAD-doped Mn complex compared to those of the undoped ones were likely to have occurred from the enhancement of the conductivity of spiro-OMeTAD under doped conditions measurements, with a two-point probe based on a glass/compact TiO_2_/HTM/Au structure, as reported previously in the literature and these are shown in Fig. 3a.^52^Fig. 6e and Table 2 show the statistical data obtained for 20 PSC devices based on the spiro-OMeTAD-doped Mn complex, which displayed excellent results, based on other PSCs. The PCE for devices with spiro-OMeTAD-doped Mn complex achieved reproducibility with an average of 15.43% ± 2.19%. The as-prepared spiro-OMeTAD-doped Mn complex showed a higher conductivity and high photocurrent densities, and the devices provided high average efficiencies when measured under 100 mW cm^−2^ illumination (AM 1.5 G).

**Table 2:** Photovoltaic parameters of PSCs, both undoped and with Mn complex-doped spiro-OMeTAD (1.0% w/w) as the HTM, measured under AM 1.5 simulated sunlight (100 mW cm^−2^ irradiance). The table includes results for the best devices

Doping ratio	*V*_oc_ (V)	*J*_sc_ (mA cm^−2^)	FF	PCE (%)
w/o	0.79 ± 0.06 (0.85)	8.58 ± 1.74 (10.32)	0.24 ± 0.17 (0.31)	2.88 ± 0.85 (3.73)
1.0% w/w	0.974 ± 0.045 (1.02)	21.521 ± 2.379 (23.97)	0.705 ± 0.085 (0.79)	15.433 ± 2.191 (17.62)

### Electrochemical impedance spectroscopy

**Fig. 7:**
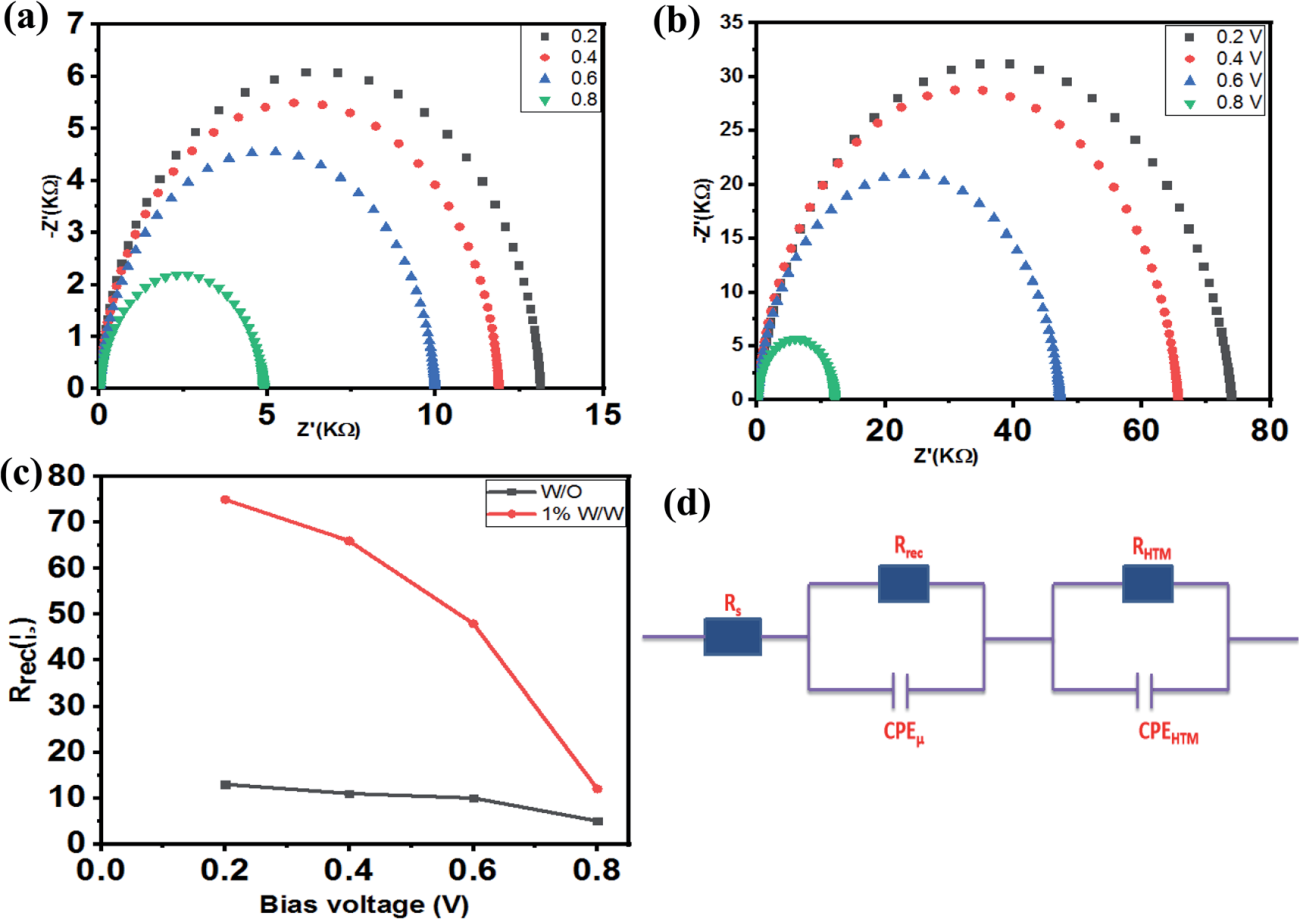
Nyquist plots of (a) spiro-OMeTAD without the Mn complex and (b) Mn complex-doped spiro-OMeTAD, tested with varied bias voltages under dark conditions. (c) A plot of the recombination resistance (*R*_rec_) *versus* bias voltage using different HTMs. (d) The equivalent circuit model for fitting the EIS; *R*_s_: series resistance, *R*_rec_: recombination resistance, *C*_PEμ_: the chemical capacitance of the film, and *R*_HTM_ and CPE_HTM_: the HTM resistance, and capacitance and extraction in the Au electrode, respectively.

To determine more of the properties of the PSC devices, EIS was used under dark conditions to gain an insight on the charge recombination in TiO_2_/(FAPbI_3_)_0.85_(MAPbBr_3_)_0.15_/HTM. Using one PSC of the spiro-OMeTAD undoped complex and one of the spiro-OMeTAD-doped Mn complexes (1.0% w/w) the charge transport parameters such as charge conductivity, recombination resistance, and chemical capacitance, were measures and the results are shown in Fig. 6a and b. Different bias voltages from 0.0 V to 0.8 V and from 106 Hz to 0.1 Hz were utilized in these measurements, and the resultant Nyquist plots are shown in Fig. 7c. As mentioned previously, the equivalent circuit model for the electrochemical impedance measurement is reported in ref. 60. The main arc represents the chemical capacitance of the film and a combination of the recombination resistance *R*_rec_. The Nyquist plot of the electrochemical impedance that represents the relationship between *R*_rec_ and the bias voltage, indicated that at the same bias voltage, the PSC device with spiro-OMeTAD-doped Mn complex (1.0% w/w) as HTM had a higher recombination resistance for applied voltage, which showed a larger *R*_rec_ than the device with a free spiro-OMeTAD based PSC as HTM, which lead to higher conductivity and provided a higher *J*_sc_, as shown in Fig. 7a and b.^53–56^

### Stability of PSCs

**Fig. 8:**
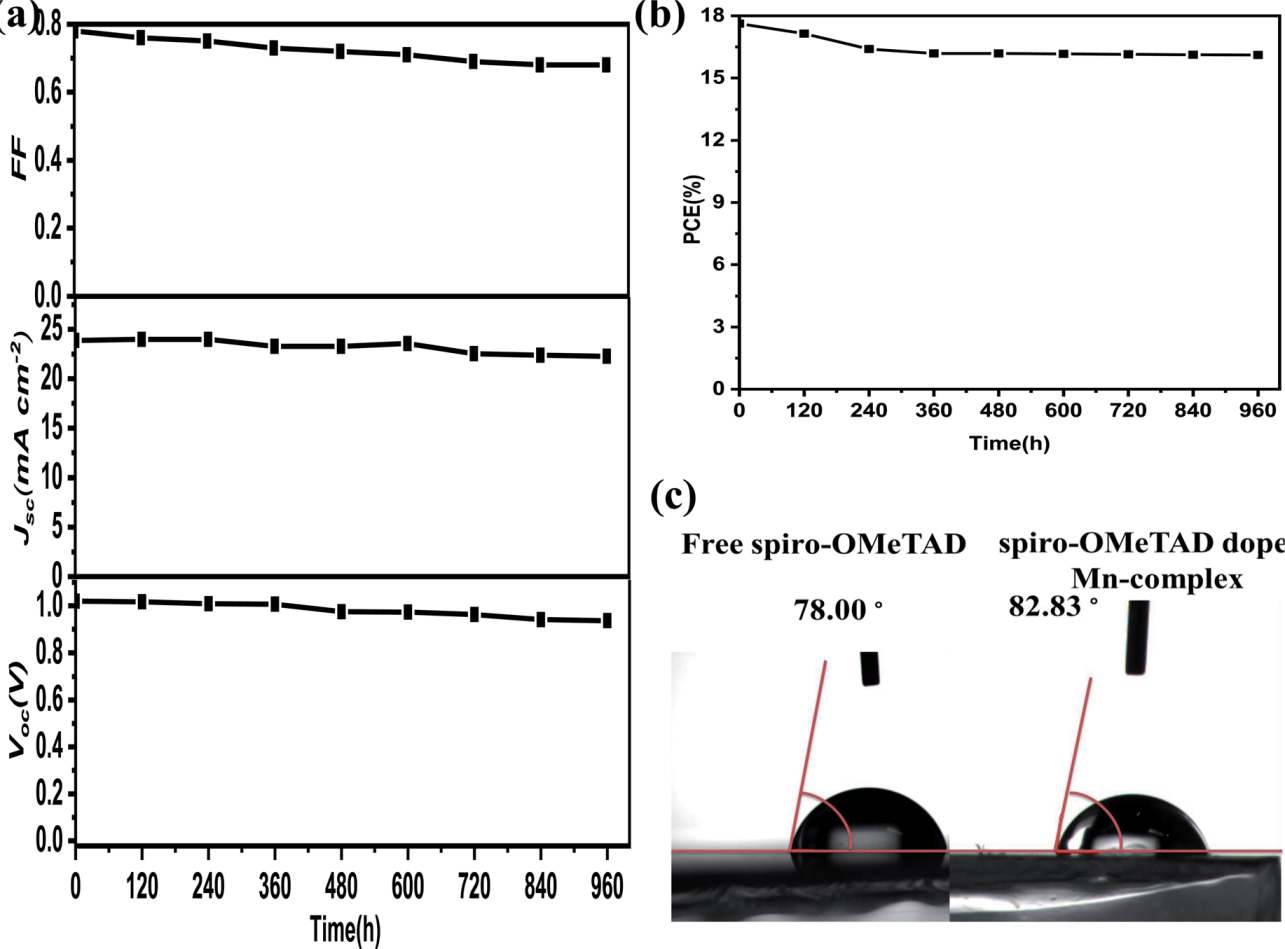
(a) Changes in the photovoltaic parameters *V*_oc_, *J*_sc_, and FF during stability measurements. (b) The PCE of a PSC based on the spiro-OMeTAD doped Mn complex as the HTM measured under AM 1.5 simulated sunlight (for PSC stability testing during the shown time period). (c) Water contact angles of free-spiro-OMeTAD and Mn-complex-doped thin films deposited on FTO.

Stability testing using the level of water penetration in undoped spiro-OMeTAD and doped Mn complex was performed. The water contact angles obtained, for undoped and doped spiro-OMeTAD with Mn complex, were 78.0° and 82.83°, respectively, and are shown in Fig. 8c. The hydrophobic properties of the Mn complex could stop water penetration into the perovskite layer and thus improve the stability of the PSCs. Furthermore, under ambient conditions in the dark with encapsulation and humidity at ∼45%, the PSCs exhibited high stability when the efficiency stability and photovoltaic parameters, were measured for 960 h, and the results are shown in Fig. 8a and b. The device showed excellent long-term stability and presented a PCE change from 17.62% to 16.16%, implying that 91.7% of its initial efficiency remained over the aging time, and that there was long-term stability for the other parameters. Stability tests revealed that the Mn complexes have a remarkably positive effect on the long-term stability of PSCs, but the Li-TFSI in the composite influences the FF and *J*_sc_ of the solar cells.

## Experimental

### Materials

All the chemicals and reagents were purchased from Sigma-Aldrich and used as received. These included *N*-methylethane-1,2-diamine (98%), CH_2_Cl_2_ (99.5%), 2-chloromethylpyridine hydrochloride (98%), sodium hydroxide (97%), manganese perchlorate (Mn(ClO_4_)_2_, 99%), PbBr_2_ (99%), HI (48% in water), HBr (48% in water), CH_3_NH_2_ (33 wt% in absolute ethanol), formamidine acetate (99%), titanium diisopropoxide bis(acetylacetonate) (75% in isopropanol, Tiacac), spiro-OMeTAD, Li-TFSI, and TBP (96%). The PbI_2_ (>98%) was obtained from TCI, and the mesoporous TiO_2_ paste (18NR-T) was purchased from Dyesol.

### Synthesis of perovskite

The NH=CHNH_3_I and CH_3_NH_3_Br (MABr) were synthesized as reported previously.^15^ The (FAPbI_3_)_1−*x*_(MAPbBr_3_)_*x*_ (*x* = 0.15) was prepared as a mixed-cation perovskite precursor solution in a glovebox, by dissolving FAI (1 M), MABr (0.2 M), PbI_2_ (1.1 M), PbBr_2_ (0.2 M) in a mixed solvent of dimethylformamide, and dimethyl sulfoxide (4 : 1, v/v), as reported previously.^53–58^

### Synthesis of (*N*-methyl-*N*,(*N*′,*N*′)-tris(2-pyridylmethyl)ethylenediamine (Me-tpen))

A solution of *N*-methylethane-1,2-diamine (2.96 g, 40 mmol) in CH_2_Cl_2_ (50 ml) was added to a solution of 2-chloromethylpyridine hydrochloride (19.66 g, 120 mmol) in water (40 ml) and gently stirred at room temperature. Then, a 9.6 M aqueous solution of NaOH (25 ml) was slowly added to the mixture within 24 h. After that, the mixture was stirred for a further 3 d. The reaction mixture was extracted three times with CH_2_Cl_2_ (3 ml each time). The combined organic phase was dried over MgSO_4_ and evaporated to dryness to produce a brown colored oil. The crude product was purified by column chromatography (basic Al_2_O_3_), and eluted with CH_2_Cl_2_ : CH_3_OH (10 : 1) (v/v) to produce a dark red colored oil. The ^1^H-NMR (CDCl_3_) data: *d* 8.44–7.12 (m, 12H, PyH), 3.90 (s, 4H), 3.74 (s, 2H), 2.66 (m, 4H), 2.25 (s, 3H). (ESI-MS (*m*/*z*)): ([[M + H]^+^]) 348.40.^49^

### Synthesis of [Mn(Me-tpen)(ClO_4_)_2_]^2+^

A solution of Me-tpen (0.132 g, 0.38 mmol) dissolved in CH_2_Cl_2_ (2 ml) was added dropwise to a solution of Mn(ClO_4_)_2_·6H_2_O (0.177 g, 0.49 mmol) in acetonitrile (CH_3_CN, 2 ml). The mixture was stirred overnight, under vacuum, and the solvent was removed to produce a pink colored powder. The crude product was recrystallized three times from CH_3_CN/Et_2_O to yield a pink colored product (0.162 g, 72%). ESI-MS (*m*/*z*): [(Mn(Me-tpen)(ClO_4_)_2_^−^)]^2+^ 501.20, [[Mn(Me-tpen)]^2+^] 201.09.^59^

### Device fabrication and characterization

The etching of the FTO was performed by using zinc powder and hydrochloric acid (2 M). Next, the etched slides were cleaned with detergent, deionized water, and ethanol. The organic residue was removed by placing the slide under UV originating from oxygen plasma for 30 min. Using spray hydrolysis, a TiO_2_ compact layer, and a compact TiO_2_ titanium di-isopropoxide bis(acetylacetonate) layer were placed on the surfaces of the cleaned FTO glasses, diluted in anhydrous ethanol at a volumetric ratio of 1 : 10 with the blocking layer (BL), and about 30–40 nm was deposited, and it was then heated at 500 °C for 30 min. Spin-coating was used to deposit TiO_2_ paste (18NR-T, Dyesol) as a mesoporous TiO_2_ layer dissolved in diluted anhydrous ethanol at a ratio of 1 : 5.5 (w/w) at 5000 rpm for 30 s, and the layers were sintered in air at 500 °C for 30 min. Using the precursor solution on mesoporous TiO_2_/BL TiO_2_/FTO substrates, the mixed-cation perovskite films were deposited *via* a two-step spin-coating procedure at 1000 rpm for 10 s and then at 5000 rpm for 20 s; 15 s after the second step was completed, 200 μl of chlorobenzene was dropped onto the substrates. The substrate was then heated on a hotplate at (100 °C) for 60 min. The substrates were cooled to room temperature, and different concentrations of spiro-OMeTAD:Mn complex were deposited on the perovskite layers at 2000 rpm for 30 min *via* the solution process. The spiro-OMeTAD solutions were prepared by dissolving 80 mg in 1 ml of chlorobenzene, 32 μl of TBP, and 17.5 μl of Li-TFSI solution (520 mg Li-TFSI in 1 ml of CH_3_CN).

Next, the Mn complex was dissolved in CH_3_CN solution at a concentration of 0.8 mg in 30 μl before adding it to the spiro-OMeTAD solution. As a comparison, Mn complexes of 0.4, 1.2, or 2.0 mg were used to optimize the concentration for use with spiro-OMeTAD as the HTM. The HTM layers were spin-coated onto the substrates and heated at (65 °C) for 15 min. Finally, a 100 nm layer of Au under a high vacuum (<4 × 10^−4^ Pa) was deposited by thermal evaporation on the top of the HTM layers. A Keithley 2400 SourceMeter was used to characterize the photocurrent–voltage, *J*–*V* of the solar cells under simulated sunlight (AM 1.5 G, 100 mW cm^2^) generated using an Oriel Sol3A solar simulator (Newport USA, Model: 94023A) under ambient conditions. A Newport calibrated standard Si reference cell (Serial No. 506/0358) was used to calibrate the light intensity. A black mask was applied to the top of the cell with a 0.09 cm^2^ circular aperture which was smaller than the active area of the square solar cell (0.20 cm^2^). The *J*–*V* curves were obtained from forwarding bias voltages to short-circuit at a scan rate of 20 mV s^−1^. The incident photo-to-current conversion efficiency (IPCE) was measured using an Oriel IQE-200 (Newport USA, Model: 94023A/PVIV-212V/IQE-AC-QTH-SI-220). Prior to measurement, a standard silicon solar cell was used as a ref. 53–58.

### Characterization

The UV-vis spectra were obtained using an Agilent 8453 spectrophotometer (Model: HP 8435, China). High resolution scanning electron microscopy (HR-SEM) was used to obtain cross-sectional images was carried out using a Nova NanoSEM 450 field emission SEM (FEI, USA). Electrocatalytic measurements and electrolysis experiments were carried out at room temperature (25 °C ± 2 °C) using a CHI 660E electrochemical analyzer (CH Instruments) in a three-electrode setup. Conductivity measurements were performed as follows.^45^ The glass substrate was cleaned with detergent, deionized water, and ethanol. The glass was placed under oxygen plasma for 30 min to remove any organic residues present. A TiO_2_ (30 nm) compact layer was deposited on the glass by spray hydrolysis, and centered under 500 °C for 30 min, and then cooled to room temperature. The HTM p-doped substrates were dissolved in different concentrations in CB, and spin coating on c-TiO_2_ was carried out in a similar way as mentioned above for the photovoltaic device. By thermal evaporation under a high vacuum (<4 × 10^−4^ Pa), 100 nm of Au was deposited on the top of the HTM films. A two-point probe evaporation setup was used to measure the linear current–voltage curves with a Keithley 2400 SourceMeter. All measurements were carried out at room temperature. Electrochemical impedance spectroscopy (EIS) was used at different applied bias voltages under dark conditions using a Zennium impedance/gain-phase analyzer (Zahner, Germany Serial No. 40037), and the scanning frequency range was from 10^6^ Hz to 0.1 Hz.

## Conclusions

In summary, using a spiro-OMeTAD-doped Mn complex as the HTM was successfully demonstrated for mesoscopic PSCs. Bulk conductivity increased under the optimal conditions by five orders of magnitude. Oxidation of spiro-OMeTAD occurred upon introducing the Mn complex, as indicated in the UV-vis spectroscopy measurements. The UV-vis spectra indicated that the oxidation of spiro-OMeTAD occurs *via* the reduction of the Mn complex, which improved the bulk conductivity. The 17.62% efficiency of the PSC was obtained based on conductivity enhancement from the increased photocurrent density and the effectiveness of the charge collection. 91.7% of the initial efficiency remained during the aging time. In contrast, two types of copper(ii) complexes,^43^, [Cu(bpm)_2_ and Cu(bpcm)_2_], have been utilized as spiro-OMeTAD p-dopants, and the devices with 8% dopant exhibited a higher efficiency of 18.5%; however, devices with Cu(bpcm)_2_-doped spiro-OMeTAD under ambient conditions with a humidity of 30–40% and a temperature of 20–25 °C, without encapsulation, displayed poor stability, and after 20 days only 75% of the initial PCE remained. For cobalt(iii) complex-based^37^ PSC devices, where they were used as the p-dopant for spiro-OMeTAD, MY11, and tris[2-(1*H*-pyrazol-1-yl)pyrimidine]cobalt(ii)bis-[bis(trifluoromethylsulfonyl)imide] as a new type of p-dopant were synthesized, and a higher PCE of 12% was displayed when MY11 (15.8%) was used as a spiro-OMeTAD p-dopant. No stability data were mentioned for these dopant-based PSCs, and the conductivities of the spiro-OMeTAD doped Cu(ii) and Co(iii) complexes were increased by two orders of magnitude only. Finally, this research showed that the spiro-OMeTAD-doped Mn complex as a HTM for PSCs could work effectively in high-performance solar cells.

## Author contributions

Mohammed Elawad: designed the project and carried out the experiments, analysis, methods, and writing – original draft. Kingsley Igenepo John: writing, review and editing. Ahmed Mahmoud Idris: writing, review and editing. Li Yang: review and editing. Yuan Gao: review and editing.

## Conflicts of interest

The authors do not have any conflicts of interest to declare.

## Supplementary Material
